# The early dFLC level after autologous stem cell transplantation can predict the long-term prognosis of in patients with light chain amyloidosis

**DOI:** 10.1007/s00277-026-07238-z

**Published:** 2026-08-11

**Authors:** Jixiang Chen, Wencui Chen, Xiaomei Wu, Jinzhou Guo, Weiwei Xu, Xianghua Huang

**Affiliations:** 1 Jinling Hospital, Affiliated Hospital of Medical School, National Clinical Research Center of Kidney Diseases, Nanjing University, Nanjing, China; 2 305 East Zhongshan Road, Nanjing, 210016 China

**Keywords:** Light chain amyloidosis, Autologous stem cell transplantation, Serum free light chain, Difference between involved and uninvolved free light chains, Prognosis

## Abstract

Recent studies show that the post-treatment difference between involved and uninvolved light chains (dFLC) predicts long-term prognosis in light chain (AL) amyloidosis. However, this issue remains underexplored in patients treated with autologous stem cell transplantation (ASCT). We retrospectively reviewed 238 patients with AL amyloidosis who underwent ASCT between 2009 and 2024. Based on day 30 post-ASCT dFLC, patients were grouped as dFLC < 10 mg/L (*n* = 101) and dFLC ≥ 10 mg/L (*n* = 137). The dFLC < 10 mg/L group demonstrated significantly higher hematologic overall response rate (ORR) and complete response rate (100% vs. 73.0%; 50.5% vs. 5.8%, both *P* < 0.001), as well as superior renal ORR (94.0% vs. 75.7%, *P* < 0.001) and cardiac ORR (78.8% vs. 48.6%, *P* = 0.004). After a median follow-up of 63 months, the dFLC < 10 mg/L group showed longer median overall survival (OS) (not reached vs. 147 months, *P* < 0.001) and prolonged major organ deterioration-progression-free survival (MOD-PFS) (54 months vs. 28 months, *P* = 0.002). Multivariate analysis identified that early post-ASCT dFLC < 10 mg/L is an independent protective factor for MOD-PFS. In conclusion, the early post-ASCT achievement of dFLC < 10 mg/L predicts deeper responses, prolonged survival, and delayed progression in patients with AL amyloidosis undergoing ASCT. Clinicaltrials.gov: NCT04210791.

## Introduction

Systemic light chain amyloidosis (AL amyloidosis) is a rare and serious plasma cell dyscrasia. The pathognomonic feature is the misfolding and aggregation of monoclonal immunoglobulin light chains into insoluble fibrils that deposit in vital organs, resulting in progressive impairment of physiological function, frequently involving the kidneys, heart, liver, and peripheral nervous system [1, 2]. Autologous stem cell transplantation (ASCT) is the first-line therapy for eligible patients with AL amyloidosis, due to its ability to rapidly eliminate clonal plasma cells and the amyloidogenic light chains they produce, leading to sustained, deep remissions [3].

In AL amyloidosis, circulating free light chains (FLCs) cause tissue damage through multiple mechanisms and are the fundamental drivers of organ dysfunction [4, 5]. Consequently, therapeutic strategies aim to achieve a rapid reduction in FLC levels to mitigate organ injury and improve clinical outcomes. While traditional hematologic response criteria have relied on reductions in serum and urine M-protein, the FLC assay, specifically the difference between involved and uninvolved light chains (dFLC), has emerged as a key tool for assessing treatment response and predicting prognosis, owing to its superior sensitivity [6–8].

While the 2012 International Consensus Guidelines established a dFLC < 40 mg/L (very good partial response, VGPR) as the therapeutic target [9], advances in treatment have since driven the pursuit of deeper responses. Building on this, Manwani et al., in a prospective study of 915 patients receiving first-line bortezomib-based therapy, demonstrated that achieving the stringent dFLC response (< 10 mg/L) at 6 months post-treatment is associated with superior long-term outcomes and improved organ function [10]. Subsequently, Muchtar et al. further proposed incorporating dFLC ≤ 10 mg/L into the criteria for hematologic complete response (CR), defining it as a stringent complete response [7]. Previous studies have predominantly evaluated the prognostic role of dFLC at 3, 6, and 12 months after chemotherapy-based regimens. In contrast, the independent prognostic significance of early (1-month) dFLC measurement following ASCT in patients with AL amyloidosis has not been systematically investigated.

To address this, we conducted a retrospective analysis of patients with AL amyloidosis who underwent ASCT at our center, aiming to evaluate the prognostic impact of the early post-ASCT dFLC measurement and, specifically, to determine whether achieving a dFLC < 10 mg/L independently predicts long-term outcomes in this population.

## Methods

### Patients and eligibility

This retrospective, single-center study included consecutive patients with renal biopsy-proven AL amyloidosis at the National Clinical Research Center of Kidney Diseases, Jinling Hospital, Nanjing, from January 2009 to December 2024. Disease staging was performed according to both the Mayo 2004 and Mayo 2012 staging systems [11, 12]. According to the Mayo 2004 staging system, patients were stratified into stages I to III using the following criteria: cardiac troponin T (cTnT) ≥ 0.035 µg/L or cardiac troponin I (cTnI) > 0.1 µg/L, and NT-proBNP ≥ 332 ng/L. For the Mayo 2012 staging system, patients were classified into stages I to IV based on three thresholds: NT-proBNP > 1800 ng/L, cTnT > 0.025 µg/L, and dFLC > 180 mg/L.

Eligible patients met the following criteria: (1) histologically confirmed AL amyloidosis by positive Congo red staining on tissue biopsy; (2) received ASCT as first-line therapy per consensus eligibility criteria. Exclusion criteria included: (1) non-AL amyloidosis or those meeting the International Myeloma Working Group (IMWG) diagnostic criteria for multiple myeloma; (2) death or loss to follow-up within 30 days post-ASCT; (3) baseline dFLC < 50 mg/L; (4) missing key prognostic data. A total of 238 patients were ultimately enrolled in this study. Relevant laboratory data were collected from all patients following ASCT, with early dFLC assessment performed approximately 30 days post-ASCT. This time point was selected for most patients had achieved hematopoietic reconstitution by one month, allowing earliest evaluation of high-dose chemotherapy effects without major post-ASCT complications.

The inclusion criteria for ASCT adhered to established consensus guidelines [13], specifically including: age < 70 years; Eastern Cooperative Oncology Group (ECOG) performance status score of 0–2; New York Heart Association (NYHA) functional class I–II; echocardiographic left ventricular ejection fraction (LVEF) > 45%; NT-proBNP < 5,000 ng/L; TnT < 0.06 µg/L; oxygen saturation > 95% while breathing ambient air; total bilirubin < 34 µmol/L (2 mg/dL); baseline systolic blood pressure > 90 mmHg; estimated glomerular filtration rate (eGFR) > 30 mL·min⁻¹·(1.73 m²)⁻¹; absence of large serous cavity effusions; and no active infection. The study was approved by ethics committees of Nanjing Jinling Hospital and was conducted according to the Declaration of Helsinki. All patients in this study have signed an informed consent form for the use of clinical data for research purposes.

### Treatment regimen

In this cohort of 238 patients, 85.7% received induction therapy prior to ASCT. Specifically, 159 patients (66.8%) received bortezomib-based regimens, 23 patients (9.7%) received daratumumab-based regimens, and 22 patients (9.2%) received immunosuppressant-based regimens. The remaining 34 patients (14.3%) proceeded directly to ASCT without induction therapy. All patients who underwent induction therapy proceeded to ASCT within 8 weeks. For stem cell mobilization, granulocyte colony-stimulating factor (G-CSF) was used as the sole agent, with a target CD34⁺ cell collection of at least 2 × 10⁶/kg. The conditioning regimen consisted of melphalan administered at either 200 mg/m² or 140 mg/m², with the dose selected based on patient age, cardiac function, and renal function. All patients received standard prophylactic antiviral therapy and proton pump inhibitors during transplantation. Transfusions of platelets and red blood cells were given based on clinical need, and other supportive measures were provided as indicated.

### Response criteria and outcome definitions

The best hematologic and organ responses achieved during first-line therapy served as key efficacy endpoints. Hematologic response, evaluated according to baseline dFLC, was defined as follows: stringent complete response (sCR) required meeting CR criteria with iFLC ≤ 20 mg/L and dFLC ≤ 10 mg/L; CR required negative serum and urine immunofixation with normal FLC ratio and levels; VGPR required dFLC < 40 mg/L; partial response (PR) required either a > 50% reduction in dFLC for patients with baseline dFLC > 50 mg/L or a decrease to < 10 mg/L for those with baseline dFLC 20–50 mg/L; no response (NR) was defined as failure to meet PR criteria without progression [14, 15].

Organ responses were evaluated based on the most recent consensus guidelines [16, 17]. Patients with a baseline NT-proBNP ≥ 650 pg/mL or BNP ≥ 150 pg/mL, and at least one post-baseline measurement, were eligible for cardiac response assessment. Cardiac response criteria were defined as follows: CR was defined as NT-proBNP ≤ 350 ng/L and BNP < 80 ng/L; VGPR was defined as a reduction in NT-proBNP of > 60%; PR was defined as a reduction in NT-proBNP of 31%–60%. Patients with baseline 24-hour urinary protein excretion > 500 mg and at least one post-baseline measurement were considered evaluable for renal response. Renal response criteria were defined as follows: CR was defined as 24-hour urine protein ≤ 200 mg/24 h with a decline in eGFR of no more than 25%; VGPR was defined as a reduction in urine protein > 60%; PR was defined as a reduction in urine protein of 31%–60%.

Overall survival (OS) was measured from the initiation of induction or first-line therapy until death from any cause. Major organ deterioration-progression-free survival (MOD-PFS) was measured from the start of treatment until the first occurrence of hematologic progression, organ progression, or death from any cause, whichever occurred first. Renal survival was measured from the start of induction or first-line therapy to the onset of dialysis dependence. Patients were followed from the start of induction or first-line therapy until the last follow-up date (December 31, 2025).

### Statistical analysis

All statistical analyses were performed using SPSS version 27.0 (IBM Corp., Armonk, NY, USA). Survival curves were generated with GraphPad Prism version 10.6 (GraphPad Software Inc., San Diego, CA, USA). Continuous variables were summarized as mean ± standard deviation for normally distributed data or as median [interquartile range (IQR)] for non-normally distributed data. Categorical variables were presented as counts and percentages. The primary endpoints were OS and MOD-PFS for the entire cohort, while secondary endpoints included the best hematologic response and best organ response rates. The Kaplan-Meier method was employed to estimate survival distributions, and the log-rank test was used to compare survival between groups. Univariate and multivariate Cox proportional hazards regression models were constructed to assess risk factors associated with MOD-PFS, with results reported as hazard ratios (HR) and corresponding 95% confidence intervals (CI). Variables included in the univariate analysis were selected based on clinical relevance and prior literature, including age, sex, Mayo stage (2004 version), number of involved organs, cardiac involvement, baseline dFLC, pre-ASCT hematologic response status, and early post-ASCT dFLC (categorized as < 10 mg/L vs. ≥10 mg/L). Variables with a P value < 0.10 in the univariate analysis were entered into the multivariate Cox proportional hazards model. All statistical tests were two-sided, and a P value < 0.05 was considered to indicate statistical significance.

## Results

### Baseline characteristics

A total of 390 patients who received ASCT at our center from January 2009 to December 2024 were retrospectively screened. After applying the exclusion criteria, 146 patients with baseline dFLC < 50 mg/L were excluded, 2 patients were excluded due to loss to follow-up or incomplete efficacy data, and 4 patients who died within 30 days post-ASCT were excluded according to the predefined criteria. Causes of early death were: transplant-related mortality (*n* = 2), progressive disease (*n* = 1), and cerebral infarction (*n* = 1). Notably, all four patients with early death had severe cardiac involvement compared with those included in the final analysis. Consequently, 238 patients were ultimately enrolled in the intention-to-treat (ITT) analysis. The baseline demographic and clinical characteristics of the study population at diagnosis are presented in Table 1.

**Table 1:** The baseline characteristics of the group

Characteristics	All patients (*N* = 238)
Age, years, median (IQR)	53 (48–58)
Gender, male, n (%)	116 (48.7)
Organ involvement, n (%)	
Kidney	238 (100.0)
Heart	110 (46.2)
Liver	9 (3.8)
Nervous system	3 (1.3)
≥ 2 organ involvement	115 (48.3)
Involved FLC, n (%)	
Lambda	207 (87.0)
Kappa	31 (13.0)
κ/λ, median (IQR)	0.13 (0.07–0.23)
iFLC, mg/L, median (IQR)	127.12 (93.27–170.5)
dFLC, mg/L, median (IQR)	108.87 (76.29-155.75)
Initial induction therapy regimen, n (%)	
Bortezomib-based regimens	159 (66.8)
Daratumumab-based regimens	23 (9.7)
Immunomodulatory-based regimens	22 (9.2)
No induction therapy	34 (14.3)
Bone Marrow plasma cells, median (IQR)	2.5 (2.0-4.5)
ECOG-PS, n (%)	
0	9 (3.8)
1	130 (55.1)
2	89 (37.7)
3	8 (3.4)
Mayo 2004 cardiac stage, n (%)	
I	116 (48.9)
II	66 (27.8)
IIIA	55 (23.2)
Mayo 2012 cardiac stage, n (%)	
I	113 (47.7)
II	79 (33.3)
III	37 (15.6)
IV	8 (3.4)
Renal stage, n (%)	
I	122 (51.3)
II	110 (46.2)
III	6 (2.5)
NT-proBNP, ng/L, median (IQR)	2591.65 (898.45-9630.5)
BNP, ng/L, median (IQR)	65.15 (24.52–211)
Troponin-T, pg/mL, median (IQR)	0.019 (0.009–0.039)
ALP, U/L, median (IQR)	65 (50–87)
Urinary proteinuria, g/24 h, median (IQR)	4.67 (2.48–7.93)
Creatinine, mg/dL, median (IQR)	0.79 (0.65–0.98)
eGFR, mL/min/1.73m^2^, mean ± SD	91.84 ± 20.92
Serum albumin, g/dL, mean ± SD	27.80 ± 6.62
Septal thickness, mm, median (IQR)	11 (10–13)
LVEF, %, mean ± SD	63.59 ± 7.81
Melphalan dose, n (%)	
140 mg/m²	66 (27.7)
200 mg/m²	158 (66.4)
Other doses (e.g., 100 mg/m²)	14 (5.9)
Pre-ASCT hematologic response	
CR, % (n)	50 (21.0)
VGPR, % (n)	74 (31.1)
PR, % (n)	23 (9.7)
NR, % (n)	91 (38.2)
After ASCT	
Early dFLC, mg/L, median (IQR)	13.75 (5.92–30.93)
Patients stratified by dFLC level, n (%)	
dFLC<10, mg/L	101 (42.4)
dFLC 10–40, mg/L	87 (36.6)
dFLC ≥ 40, mg/L	50 (21.0)

Abbreviations: *IQR* interquartile range, *FLC* free light chain, *iFLC* involved free light chain, *dFLC* difference between involved and uninvolved free light chains,* ECOG-PS* Eastern Cooperative Oncology Group performance status score, *eGFR* estimated glomerular filtration rate, *NT-proBNP* N-terminal pro-B-type natriuretic peptide, *BNP* brain natriuretic peptide, *ALP* alkaline phosphatase, *ASCT* autologous stem cell transplantation, *LVEF* left ventricular ejection fraction

The median age was 53 years (IQR: 48–58), and 48.7% of patients were male. The median baseline dFLC was 108.8 mg/L (IQR: 76.29–155.75). Renal involvement was present in all patients, whereas cardiac, hepatic, and peripheral nervous system involvement were observed in 46.2%, 3.8%, and 1.3% of patients, respectively. Multiorgan involvement (≥ 2 organs) was documented in 48.3% of cases. The median early post-ASCT dFLC was 13.75 mg/L (IQR: 5.92–30.93), with the measurement performed at a median of 29 days post-ASCT (range: 19–46 days). Prior to ASCT, the best hematologic responses achieved were as follows: CR in 50 patients (21.0%), VGPR in 74 (31.1%), PR in 23 (9.7%), and NR in 91 (38.2%). Based on previously reported prognostic cutoffs, patients were divided into two groups: dFLC < 10 mg/L (*n* = 101, 42.4%) and dFLC ≥ 10 mg/L (*n* = 137, 57.6%). To better delineate prognostic differences associated with varying depths of response, the dFLC ≥ 10 mg/L group was further subdivided into two categories: dFLC 10–40 mg/L (*n* = 87, 36.6%) and dFLC ≥ 40 mg/L (*n* = 50, 21.0%).

### Hematologic and organ responses

The ITT analysis of hematologic responses is presented in Tables 2 and 3. In the early post-ASCT assessment, the dFLC < 10 mg/L group had significantly higher rates of sCR and CR than the dFLC ≥ 10 mg/L group (sCR: 28.7% vs. 0%; CR: 50.5% vs. 5.8%; both *P* < 0.001). The ORR was also significantly higher in the dFLC < 10 mg/L group (100% vs. 73.0%, *P* < 0.001). At 3 months post-ASCT, the sCR and CR rates remained significantly higher in the dFLC < 10 mg/L group compared with the dFLC ≥ 10 mg/L group (sCR: 34.7% vs. 0.7%; CR: 53.5% vs. 7.3%; both *P* < 0.001). A similar pattern was observed at 6 months post-ASCT (sCR: 35.6% vs. 1.5%; CR: 52.5% vs. 5.8%; both *P* < 0.001). Regarding the best hematologic response achieved during first-line therapy, the dFLC < 10 mg/L group again demonstrated superior sCR and CR rates compared with the dFLC ≥ 10 mg/L group (sCR: 43.6% vs. 0%; CR: 70.3% vs. 13.9%; both *P* < 0.001). The ORR was also significantly higher in the dFLC < 10 mg/L group (100% vs. 81%, *P* < 0.001).

**Table 2:** The hematologic response in first-line treatment

	1 months			3 months			6 months		
	dFLC<10 mg/L (*N* = 101)	dFLC ≥ 10 mg/L (*N* = 137)	*P* value	dFLC<10 mg/L (*N* = 101)	dFLC ≥ 10 mg/L (*N* = 137)	*P* value	dFLC<10 mg/L (*N* = 101)	dFLC ≥ 10 mg/L (*N* = 137)	*P* value
sCR, % (n)	29 (28.7)	0	**<0.001**	35 (34.7)	1 (0.7)	**<0.001**	36 (35.6)	2 (1.5)	**<0.001**
CR, % (n)	51 (50.5)	8 (5.8)	**<0.001**	54 (53.5)	10 (7.3)	**<0.001**	53 (52.5)	8 (5.8)	**<0.001**
VGPR, % (n)	50 (49.5)	80 (58.4)	0.173	47 (46.5)	83 (60.6)	**0.031**	48 (47.5)	85 (62.0)	**0.026**
PR, % (n)	0	12 (8.8)	**0.002**	0	13 (9.5)	**0.001**	0	13 (9.5)	**0.001**
NR, % (n)	0	37 (27.0)	**<0.001**	0	31 (22.6)	**<0.001**	0	31 (22.6)	**<0.001**
ORR, % (n)	100 (100)	100 (73.0)	**<0.001**	101 (100)	106 (77.4)	**<0.001**	101 (100)	106 (77.4)	**<0.001**

Abbreviations: *dFLC* difference between involved and uninvolved free light chains, *ORR* overall response, *CR* complete hematological response, *VGPR* very good partial response, *PR* partial response, *NR* no response

Regarding organ responses (Table 3), 236 patients were evaluable for renal response. At the assessment of best renal response during first-line therapy, the dFLC < 10 mg/L group demonstrated a significantly higher ORR compared to the dFLC ≥ 10 mg/L group (94.0% vs. 75.7%, *P* < 0.001), as well as a superior CR rate (42.0% vs. 16.2%, *P* < 0.001). For cardiac response, 103 patients were evaluable. The dFLC < 10 mg/L group also showed a significantly higher ORR (78.8% vs. 48.6%, *P* = 0.004) and CR rate (45.5% vs. 15.7%, *P* = 0.001) compared to the dFLC ≥ 10 mg/L group.

**Table 3:** The best hematologic response and best organ response during first-line treatment between the two groups

	Hematology			Renal			Cardiac		
	dFLC<10 mg/L (*N* = 101)	dFLC ≥ 10 mg/L (*N* = 137)	*P* value	dFLC<10 mg/L (*N* = 100)	dFLC ≥ 10 mg/L (*N* = 136)	*P* value	dFLC<10 mg/L (*N* = 33)	dFLC ≥ 10 mg/L (*N* = 70)	*P* value
sCR, % (n)	44 (43.6)	0	**<0.001**	-	-	-	-	-	-
CR, % (n)	71 (70.3)	19 (13.9)	**<0.001**	42 (42.0)	22 (16.2)	**<0.001**	15 (45.5)	11 (15.7)	**0.001**
VGPR, % (n)	30 (29.7)	85 (62.0)	**<0.001**	42 (42.0)	51 (37.5)	0.485	9 (27.3)	20 (28.6)	0.891
PR, % (n)	0	7 (5.1)	**0.021**	10 (10.0)	30 (22.1)	**0.015**	2 (6.1)	3 (4.3)	0.696
NR, % (n)	0	26 (19.0)	**<0.001**	6 (6.0)	33 (24.3)	**<0.001**	7 (21.2)	36 (51.4)	**0.004**
ORR, % (n)	101 (100)	111 (81.0)	**<0.001**	94 (94.0)	103 (75.7)	**<0.001**	26 (78.8)	34 (48.6)	**0.004**

Abbreviations: *dFLC* difference between involved and uninvolved free light chains, *ORR* overall response, *CR* complete hematological response, *VGPR* very good partial response, *PR* partial response, *NR* no response

### Survival and progression

The median follow-up time for the entire cohort was 63 months (IQR: 31–110 months). During follow-up, a total of 59 patients (24.7%) succumbed to death. In the dFLC < 10 mg/L group, 8 patients (13.5% of group) died, including 4 (50.0%) due to disease progression, 1 (12.5%) due to rectal cancer, 1 (12.5%) due to pulmonary infection, and 2 (25.0%) due to unknown causes. In the dFLC ≥ 10 mg/L group, 51 patients (86.4% of group) died, including 22 (43.1%) due to disease progression, 14 (27.4%) due to heart failure, 5 (9.8%) due to organ hemorrhage (including renal hemorrhage, cerebral hemorrhage, and gastrointestinal bleeding), 3 (5.8%) due to transplant-related mortality, 2 (3.9%) due to multiple organ dysfunction syndrome (MODS), 1 (2.0%) due to respiratory failure, and 4 (7.8%) due to unknown causes.

**Fig. 1:**
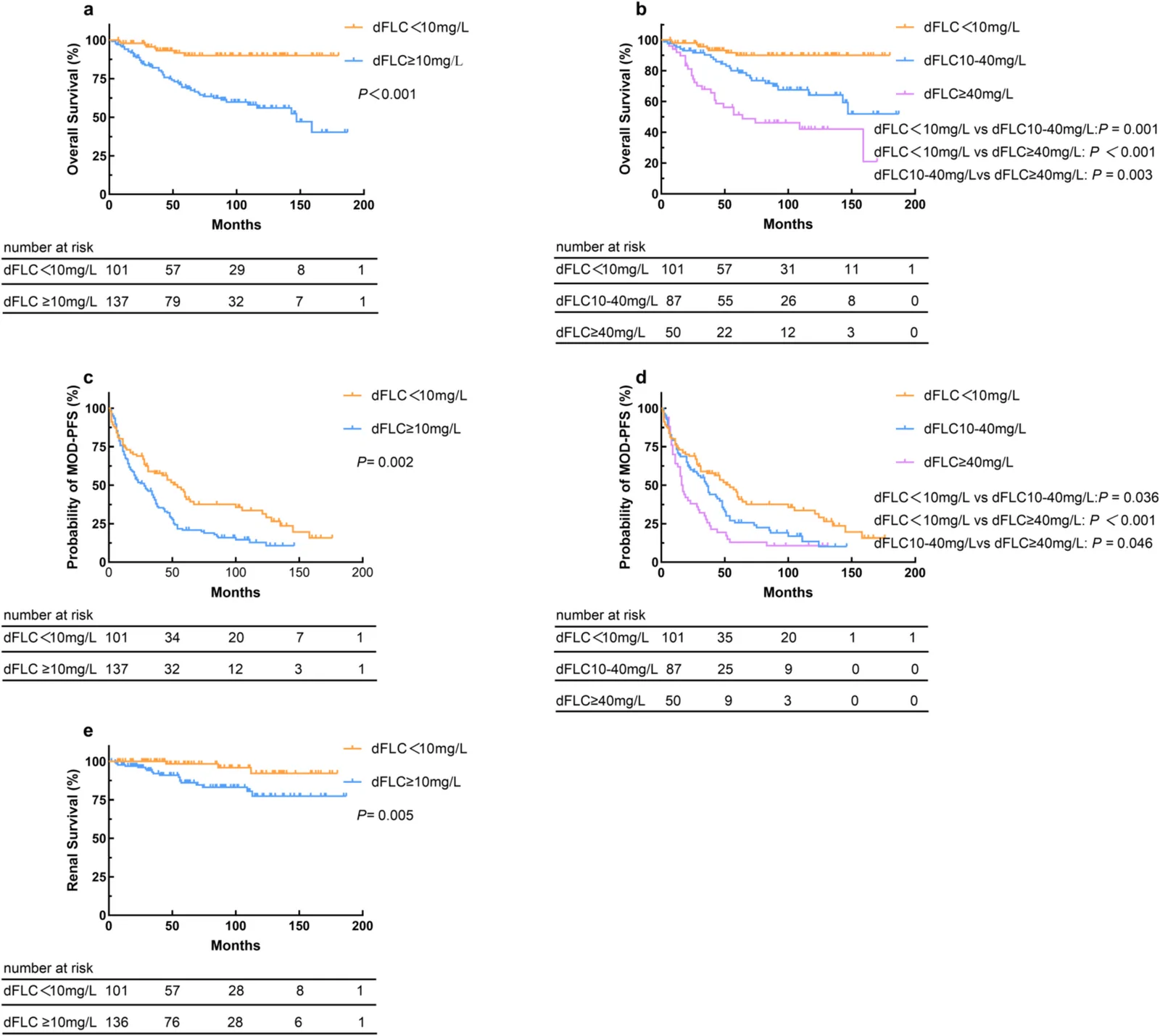
Kaplan-Meier curves depicting Overall Survival (OS) and Major Organ Deterioration Progression-Free Survival (MOD-PFS). (**a**) The OS in the dFLC < 10 mg/L group and the dFLC ≥ 10 mg/L group. (**b**) The OS in the dFLC < 10 mg/L, dFLC 10–40 mg/L, and dFLC ≥ 40 mg/L groups. (**c**) The MOD-PFS in the dFLC < 10 mg/L group and the dFLC ≥ 10 mg/L group. (**d**) The MOD-PFS in the dFLC < 10 mg/L, dFLC 10–40 mg/L, and dFLC ≥ 40 mg/L groups. (**e**) The renal survival in the dFLC < 10 mg/L group and the dFLC ≥ 10 mg/L group. Abbreviations: dFLC difference between involved and uninvolved free light chains, OS overall survival, MOD-PFS major organ deterioration progression-free survival

Kaplan-Meier survival analysis is shown in Fig. 1. The median OS was not reached in the dFLC < 10 mg/L group, compared to 147 months in the dFLC ≥ 10 mg/L group, with a statistically significant difference between groups (*P* < 0.001, Fig. 1a). Further analysis revealed a significant downward trend in survival with increasing dFLC levels, with OS in the dFLC < 10 mg/L group being significantly superior to that in both the dFLC 10–40 mg/L and dFLC ≥ 40 mg/L groups (*P* ≤ 0.001, Fig. 1b). Regarding MOD-PFS, the median MOD-PFS was 54 months in the dFLC < 10 mg/L group, which was significantly longer than the 28 months observed in the dFLC ≥ 10 mg/L group (*P* = 0.002, Fig. 1c). Additionally, it was also significantly superior to the other two subgroups (Fig. 1d). Although the median renal survival was not reached in either group, the dFLC < 10 mg/L group demonstrated significantly higher renal survival rates compared to the dFLC ≥ 10 mg/L group (*P* = 0.005, Fig. 1e). The 5-year OS rates were 98.4% and 90.0% for the dFLC < 10 mg/L and dFLC ≥ 10 mg/L groups, respectively, with corresponding 5-year renal survival rates of 85.4% and 64.0%.

### Subgroup analysis

**Fig. 2:**
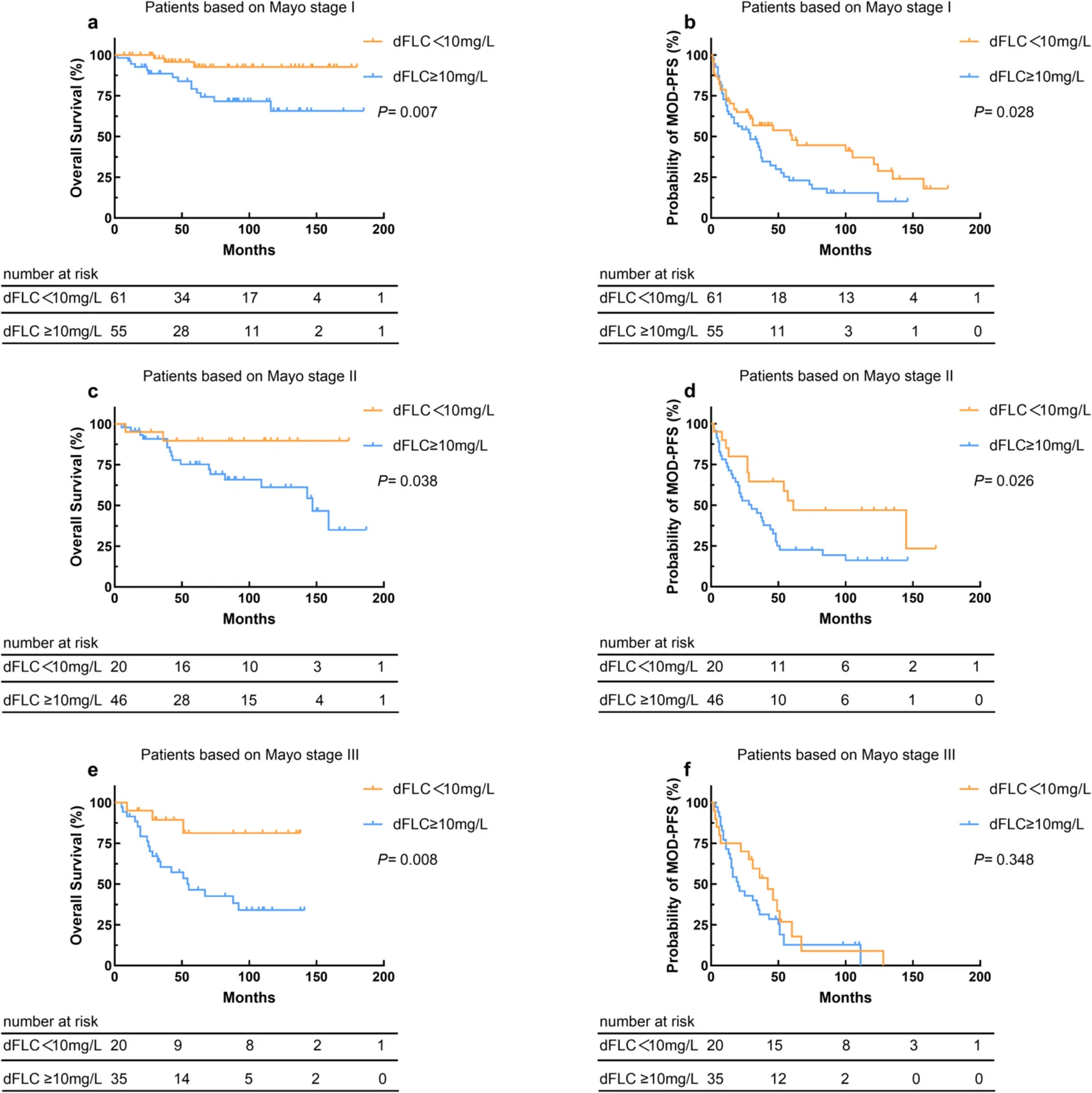
Kaplan-Meier curves for OS and MOD-PFS stratified by Mayo stage. (**a**) The OS in Mayo stage I patients: dFLC < 10 mg/L vs. dFLC ≥ 10 mg/L. (**b**) The MOD-PFS in Mayo stage I patients: dFLC < 10 mg/L vs. dFLC ≥ 10 mg/L. (**c**) The OS in Mayo stage II patients: dFLC < 10 mg/L vs. dFLC ≥ 10 mg/L. (**d**) The MOD-PFS in Mayo stage II patients: dFLC < 10 mg/L vs. dFLC ≥ 10 mg/L. (**e**) The OS in Mayo stage IIIA patients: dFLC < 10 mg/L vs. dFLC ≥ 10 mg/L. (**f**) The MOD-PFS in Mayo stage IIIA patients: dFLC < 10 mg/L vs. dFLC ≥ 10 mg/L. Abbreviations: dFLC difference between involved and uninvolved free light chains, OS overall survival, MOD-PFS major organ deterioration progression-free survival

Patients were stratified according to the Mayo 2004 staging system to evaluate survival outcomes across different risk categories. The results showed that among patients in the stage I, II, and IIIA subgroups, OS was significantly longer in those with dFLC < 10 mg/L compared to those with dFLC ≥ 10 mg/L (Fig. 2). For MOD-PFS, a significant improvement in the dFLC < 10 mg/L group was primarily observed in patients with cardiac stage I and II disease.

**Fig. 3:**
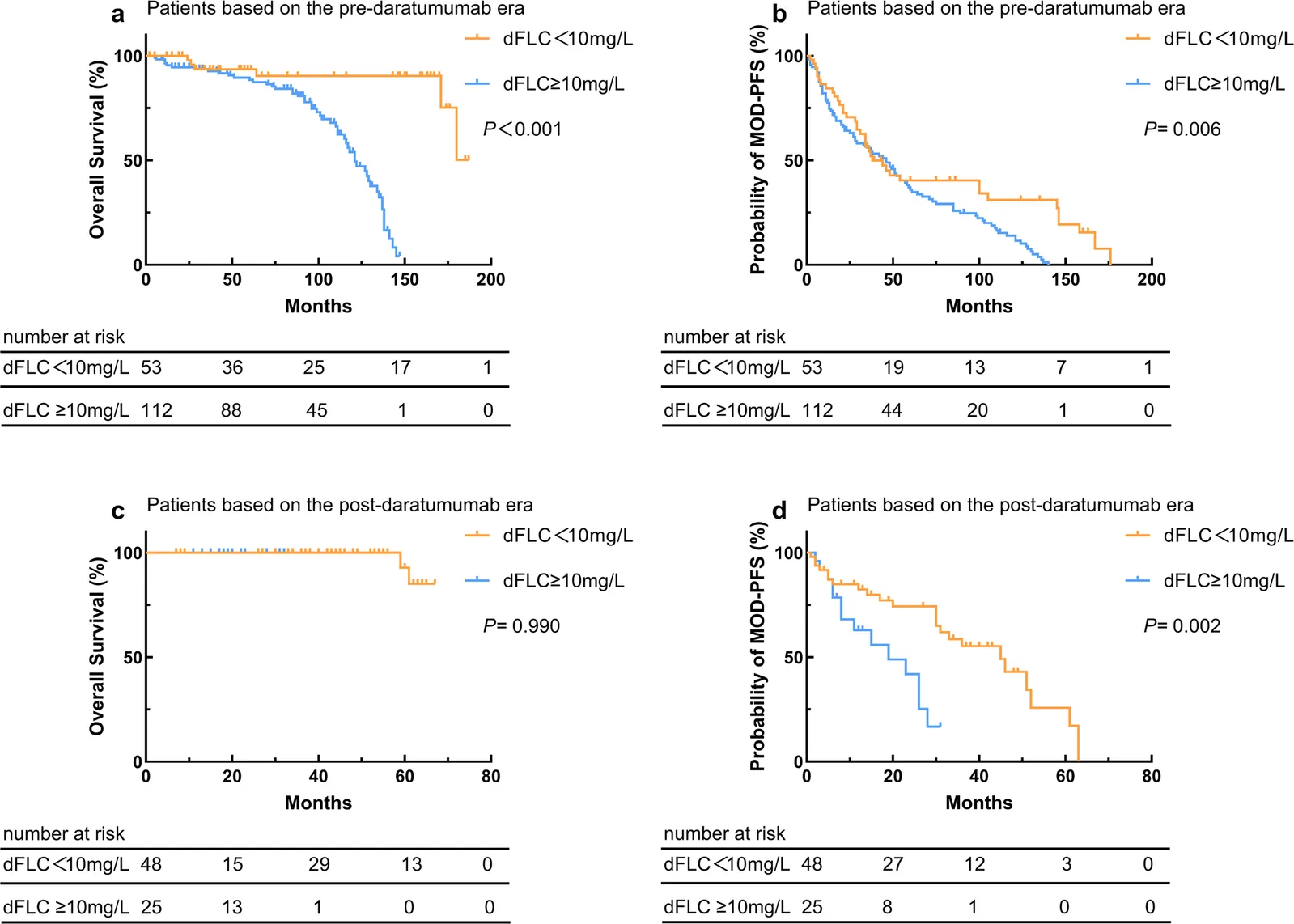
Kaplan-Meier curves for OS and MOD-PFS stratified by the addition of daratumumab (i.e., pre-daratumumab vs. post-daratumumab era). (**a**) The OS in the pre-daratumumab era-based patient cohort: dFLC < 10 mg/L vs. dFLC ≥ 10 mg/L. (**b**) The MOD-PFS in the pre-daratumumab era-based patient cohort: dFLC < 10 mg/L vs. dFLC ≥ 10 mg/L. (**c**) The OS in the post-daratumumab era-based patient cohort: dFLC < 10 mg/L vs. dFLC ≥ 10 mg/L. (**d**) The MOD-PFS in the post-daratumumab era-based patient cohort: dFLC < 10 mg/L vs. dFLC ≥ 10 mg/L

To minimize potential confounding bias due to temporal changes in treatment strategies, we compared the outcomes of patients enrolled in the early period (2009–2019, *n* = 165) and the late period (2020–2024, *n* = 73) (Fig. 3). These two eras roughly correspond to the pre-daratumumab and post-daratumumab periods at our center. In both eras, early post-ASCT dFLC < 10 mg/L was significantly associated with longer MOD-PFS (2009–2019: *P* = 0.006, Figs. 3b and 2020–2024: *P* = 0.002, Fig. 3d). However, in the post-daratumumab era, no statistically significant difference in OS was observed between the dFLC < 10 mg/L and dFLC ≥ 10 mg/L groups, although a significant difference in MOD-PFS between the two groups persisted.

### Analysis of risk factors

Univariate and multivariate analyses of MOD-PFS for all patients are presented in Table 4. The univariate analysis revealed that early post-ASCT dFLC ≥ 40 mg/L and dFLC 10–40 mg/L were significantly correlated with an elevated risk of disease progression. Additionally, elevated troponin-T levels at diagnosis and Mayo stage IIIA were identified as risk factors for MOD-PFS. Conversely, early post-ASCT dFLC < 10 mg/L and pre-ASCT hematologic response status served as protective factors for MOD-PFS. Multivariate analysis confirmed that early post-ASCT dFLC < 10 mg/L remained an independent protective factor (HR = 0.691, 95% CI: 0.498–0.961, *P* = 0.028).

**Table 4:** Univariate and multivariate analysis of risk factors for MOD-PFS

	Univariate analysis			Multivariate analysis		
Variables	Hazard Ratio	95% CI	*P* value	Hazard Ratio	95% CI	*P* value
Age, years	1.001	0.982–1.020	0.912	-	-	-
Gender (male)	1.204	0.891–1.627	0.227	-	-	-
Baseline dFLC, mg/L	0.999	0.998-1.000	0.172	-	-	-
Initial induction therapy regimen						
Bortezomib-based regimens	0.812	0.591–1.116	0.199			
Daratumumab-based regimens	1.030	0.521–2.038	0.932			
Immunomodulatory-based regimens	1.433	0.907–2.265	0.123			
No induction therapy	1.075	0.716-1/615	0.727			
Cardiac involvement	1.166	0.865–1.574	0.314			
Involvement of at least two organs	1.169	0.867–1.578	0.306			
Bone Marrow plasma cells, %	0.974	0.928–1.021	0.269			
lg (troponin-T)	1.597	1.108–2.304	**0.012**	1.429	0.835–2.445	0.193
lg (NT-proBNP)	0.898	0.704–1.147	0.389	-	-	-
Urinary proteinuria, g/24 h	1.013	0.976–1.051	0.505	-	-	-
Serum albumin, g/dL	0.986	0.964–1.010	0.250	-	-	-
κ/λ	0.990	0.970–1.010	0.315	-	-	-
Mayo2004IIIA	1.424	1.014–1.999	**0.041**	1.056	0.644–1.734	0.828
Creatinine, mg/dL	1.011	0.990–1.033	0.314	-	-	-
eGFR, mL/min/1.73m^2^	0.999	0.992–1.006	0.729	-	-	-
pre-ASCT hematologic response	0.694	0.514–0.937	**0.017**	0.766	0.562–1.044	0.091
After ASCT				-	-	-
dFLC<10 mg/L	0.608	0.443–0.834	**0.002**	0.691	0.498–0.961	**0.028**
dFLC 10-40 mg/L	1.448	1.020–2.057	**0.039**	-	-	-
dFLC≥40 mg/L	2.085	1.403–3.099	**<0.001**	-	-	-

Abbreviations: *CI* confidence intervals, *dFLC* difference between involved and uninvolved free light chains, *NT-proBNP* N-terminal pro-B-type natriuretic peptide, *ASCT* autologous stem cell transplantation

## Discussion

FLC measurements are essential for the diagnosis, prognostic stratification, and evaluation of treatment response in AL amyloidosis [18, 19]. This study provides novel evidence that an early post-ASCT dFLC < 10 mg/L serves as a reliable predictor of favorable long-term outcomes, including both OS and organ response, in patients with AL amyloidosis undergoing ASCT. Meanwhile, 42.4% of patients achieved dFLC < 10 mg/L during the early post-ASCT assessment and demonstrated favorable long-term outcomes. This finding further indicates that ASCT remains capable of achieving deep and durable responses.

Our study showed that patients achieving dFLC < 10 mg/L had significantly higher CR rates, with a renal response rate of up to 94% and significantly improved long-term renal survival. Multivariate analysis further confirmed that dFLC < 10 mg/L is an independent protective factor for MOD-PFS. These findings are consistent with previous studies. In recent years, research on the prognostic value of dFLC has advanced substantially, with accumulating evidence supporting 10 mg/L as a more precise threshold for risk stratification. Dittrich et al. first proposed that in patients with baseline dFLC 20–50 mg/L, achieving dFLC < 10 mg/L at 3 months post-treatment could accurately predict OS and renal survival [20]. Furthermore, Manwani et al. [10] and Shen et al. [21] confirmed in Western and Chinese cohorts, respectively, that patients achieving a “stringent dFLC response” (dFLC < 10 mg/L) had significant advantages in OS, time to next treatment (TNT), and cardiac response rates. The unique value of the present study lies in extending this response criterion, originally established in chemotherapy-treated populations, to the ASCT setting, with results consistent with previous reports.

In our study, patients achieving dFLC < 10 mg/L had significantly superior MOD-PFS than those with dFLC 10–40 mg/L or dFLC ≥ 40 mg/L. Univariate analysis showed that both dFLC ≥ 40 mg/L and dFLC 10–40 mg/L were associated with an increased risk of disease progression. Palladini et al. previously introduced the concept of “high-risk dFLC progression” in 2018, proposing a threshold of dFLC > 20 mg/L, which represents a > 20% increase from baseline and a > 50% increase from the best prior response, as an early warning indicator for initiating salvage therapy [22]. A recent study from our center further investigated risk factors for relapse after ASCT and confirmed that dFLC > 10 mg/L at 6 months post-transplant was an independent risk factor for disease relapse (HR = 2.15, *P* = 0.02) [23]. Based on our findings and those of others, we propose that even among patients achieving VGPR, those with dFLC persisting in the 10–40 mg/L range remain at risk for long-term organ deterioration and disease progression. These patients may still harbor residual secreting plasma cell clones producing FLCs capable of sustaining organ damage. Furthermore, our study demonstrated that patients in the dFLC ≥ 10 mg/L group had a lower probability of achieving sCR even after receiving ASCT. Therefore, for these patients, clinical observation alone is not sufficient; appropriate consolidation therapy should be considered to further reduce dFLC levels, delay disease progression, and ultimately improve long-term survival.

Subgroup analysis demonstrated that, in terms of OS, patients achieving dFLC < 10 mg/L exhibited superior long-term survival regardless of Mayo stage. However, for MOD-PFS, this benefit was primarily confined to patients with Mayo stage I and II disease, with no significant improvement observed in those with stage IIIA. Kastritis et al. similarly reported that achieving a deep hematologic response (dFLC < 10 mg/L) at 1-month post-treatment was associated with significant OS improvement across all Mayo stages, while also emphasizing that patients achieving a deep response at 3 months still had inferior long-term outcomes compared to those who achieved it by 1 month [24]. These findings further underscore that the early achievement of dFLC < 10 mg/L is closely associated with superior long-term survival. Nevertheless, for patients with Mayo stage IIIA disease, the risk of organ failure persists even with early attainment of dFLC < 10 mg/L, highlighting the need for continued aggressive optimization of therapeutic strategies in this subgroup. Further stratified analysis by treatment era demonstrated that dFLC < 10 mg/L was significantly associated with longer MOD-PFS in the pre-daratumumab era, and this trend persisted in the post-daratumumab era. However, in the post-daratumumab era, no statistically significant difference in OS was observed between the dFLC < 10 mg/L and dFLC ≥ 10 mg/L groups. This finding may be attributable to the widespread use of novel agents such as daratumumab, which have significantly improved OS, as well as the relatively shorter follow-up duration in this subgroup. Consequently, early post-ASCT dFLC remains a reliable prognostic indicator, although its predictive value for OS has diminished in the era of novel therapies.

This study has several strengths, including a relatively large sample size, long follow-up duration, and well-defined primary endpoints. To the best of our knowledge, this is the first study specifically evaluating the prognostic value of the first dFLC measurement performed at 1-month post-ASCT in AL amyloidosis. Nevertheless, several limitations should be acknowledged. First, as a single-center retrospective study, inherent selection bias cannot be completely ruled out and may have impacted the results. Second, the exclusion of patients who died within 30 days post-ASCT may have introduced survival bias. Third, because this study required a baseline dFLC > 50 mg/L, the findings may not be generalizable to patients with extremely low clonal burden. Fourth, our cohort was predominantly characterized by renal involvement, with relatively mild cardiac involvement; therefore, the results may not apply to patients with advanced cardiac disease. Fifth, the treatment landscape underwent substantial changes during the study period from 2009 to 2024, particularly with the introduction of daratumumab-based regimens in later years. Although our subgroup analysis suggested that the prognostic value of early dFLC remained largely consistent across different eras, residual confounding due to period effects cannot be entirely excluded. Finally, dFLC assays measure all FLCs of the same isotype as those of the amyloid precursor protein, rather than specifically detecting the amyloidogenic clone. Mass spectrometry-based methods may offer greater precision in the future [25].

The findings of this study have important implications for clinical follow-up. In the future, leveraging the concept of the “Internet of Surgical Things” proposed by Mulita et al., wearable devices could be employed for remote cardiac function monitoring in patients, facilitating the early detection of organ progression [26]. Additionally, the 3D printing technology described by Anagnostopoulos et al. can generate individualized organ models to help assess the extent of cardiac or renal involvement [27].

## Conclusions

In conclusion, this study confirms that the threshold of dFLC < 10 mg/L remains a reliable prognostic indicator in patients with AL amyloidosis undergoing ASCT. We believe that, for eligible patients, ASCT can still provide deep and durable responses, and early post-ASCT dFLC assessment is valuable for guiding subsequent treatment decisions, including the need for post-ASCT consolidation or maintenance therapy. Patients who achieve dFLC < 10 mg/L early post-ASCT exhibit superior hematologic and organ responses, which translate into longer survival and effective delay of disease progression. Based on our findings, we recommend that clinicians assess dFLC levels approximately 30 days after ASCT in patients with AL amyloidosis. An early post-ASCT dFLC < 10 mg/L indicates a favorable long-term prognosis. Conversely, dFLC ≥ 10 mg/L should prompt closer monitoring and consideration of early post-ASCT consolidation or maintenance therapy.

## Data Availability

The datasets used and analyzed during the current study are available from the corresponding author on reasonable request.
